# Biomodulatory Treatment With Azacitidine, All-*trans* Retinoic Acid and Pioglitazone Induces Differentiation of Primary AML Blasts Into Neutrophil Like Cells Capable of ROS Production and Phagocytosis

**DOI:** 10.3389/fphar.2018.01380

**Published:** 2018-11-27

**Authors:** Sebastian Klobuch, Tim Steinberg, Emanuele Bruni, Carina Mirbeth, Bernhard Heilmeier, Lina Ghibelli, Wolfgang Herr, Albrecht Reichle, Simone Thomas

**Affiliations:** ^1^Department of Internal Medicine III, Hematology and Oncology, University Hospital Regensburg, Regensburg, Germany; ^2^Department of Biology, University of Rome Tor Vergata, Rome, Italy; ^3^Regensburg Center for Interventional Immunology, University Hospital Regensburg, Regensburg, Germany; ^4^Department of Oncology and Hematology, Hospital Barmherzige Brueder, Regensburg, Germany

**Keywords:** azacitidine, all-*trans* retinoic acid, pioglitazone, acute myeloid leukemia, differentiation

## Abstract

Effective and tolerable salvage therapies for elderly patients with chemorefractory acute myeloid leukemia (AML) are limited and usually do not change the poor clinical outcome. We recently described in several chemorefractory elderly AML patients that a novel biomodulatory treatment regimen consisting of low-dose azacitidine (AZA) in combination with PPARγ agonist pioglitazone (PGZ) and all-*trans* retinoic acid (ATRA) induced complete remission of leukemia and also triggered myeloid differentiation with rapid increase of peripheral blood neutrophils. Herein, we further investigated our observations and comprehensively analyzed cell differentiation in primary AML blasts after treatment with ATRA, AZA, and PGZ *ex vivo*. The drug combination was found to significantly inhibit cell growth as well as to induce cell differentiation in about half of primary AML blasts samples independent of leukemia subtype. Notably and in comparison to ATRA/AZA/PGZ triple-treatment, effects on cell growth and myeloid differentiation with ATRA monotherapy was much less efficient. Morphological signs of myeloid cell differentiation were further confirmed on a functional basis by demonstrating increased production of reactive oxygen species as well as enhanced phagocytic activity in AML blasts treated with ATRA/AZA/PGZ. In conclusion, we show that biomodulatory treatment with ATRA/AZA/PGZ can induce phenotypical and functional differentiation of primary AML blasts into neutrophil like cells, which aside from its antileukemic activity may lower neutropenia associated infection rates in elderly AML patients *in vivo*. Clinical impact of the ATRA/AZA/PGZ treatment regimen is currently further investigated in a randomized clinical trial in chemorefractory AML patients (NCT02942758).

## Introduction

Clinical outcome of elderly AML patients refractory to intensive chemotherapy is poor, especially when allogeneic stem cell transplantation is not feasible due to comorbidities. Therefore, there is an urgent clinical need for alternative treatment regimens, which are effective and tolerable.

We recently described a small series of elderly AML patients treated with a novel biomodulatory drug combination consisting of low-dose AZA, the peroxisome proliferator-activated receptor γ agonist PGZ, and ATRA (Thomas et al., 2015). Beside a surprisingly high response rate (four of five patients achieved complete remission), we observed in three out of five patients a strong increase of neutrophilic granulocytes in peripheral blood within a few days after start of therapy, which is in contrast to standard dose azacitidine therapy, where neutropenia is a frequent drug-related adverse event (Dombret et al., 2015). We further analyzed neutrophilic granulocytes from peripheral blood of ATRA/AZA/PGZ treated patients and found the same AML-associated mutations originally described in patient’s AML blasts at primary diagnosis, suggesting that biomodulatory therapy with ATRA/AZA/PGZ induced differentiation in AML cells (Thomas et al., 2015).

Induction of cell differentiation is well-described for APL treated with ATRA alone or in combination with ATO (Lo-Coco et al., 2013). However, beneficial effects of ATRA in non-APL subtypes were moderate when combined with intensive induction chemotherapy and limited to patients with ELN favorable-risk (Schlenk et al., 2016). Although clinical phase I/II trials evaluating ATRA in combination with hypomethylating agents (e.g., azacitidine, decitabine) and the histone deacetylase inhibitor valproic acid showed encouraging safety and response data in elderly patients with high-risk MDS and AML, there was no evidence for the restoration of ATRA-induced cell differentiation during therapy (Soriano et al., 2007; Lübbert and Kuendgen, 2015). To further increase the effect of ATRA and AZA combination we included PGZ, as PGZ was described as potential modulator in myeloid malignancies by acting on transcription factors involved in apoptosis and cell differentiation (Konopleva et al., 2004; Saiki et al., 2006).

Neutropenia associated bacterial and fungal infections are major complications in AML patients and have a significant impact on the clinical course (e.g., more hospital admissions) as well as on the outcome of patients (Döhner et al., 2010). Induction of myeloid differentiation leading to an early increase of neutrophils already during therapy might therefore decrease the risk for serious infectious complications. However, so far it is unclear whether neutrophils differentiated from primary AML blasts are able to combat pathogens. We herein describe for the first time that biomodulatory treatment of primary AML blasts with ATRA/AZA/PGZ not merely induces morphological changes in primary AML blasts but also promotes their differentiation into granulocytes capable of phagocytosis and reactive oxygen species (ROS) production.

## Materials and Methods

### Culture of Leukemia Cells

AML cell line HL-60 was provided by Dr. M. Rehli, University Hospital Regensburg. MV4-11 and MOLM-13 cell lines were kindly provided by Dr. M. Hudecek, University Hospital Würzburg. All cell lines were routinely tested for mycoplasma contamination. Primary AML blasts were isolated from peripheral blood of patients who showed more than 70% leukemia blasts within total white blood cells at primary diagnosis. Blood samples were drawn before start of leukemia therapy after written informed consent in accordance with the Declaration of Helsinki and upon approval of the study by the ethics committee of the University Regensburg. HL-60 cells were cultured in RPMI 1640 medium (Thermo Fisher Scientific, Waltham, MA, United States) supplemented with 10% FCS (PAA Laboratories, Pasching, Germany), 2% Penicillin-Streptomycin (Life Technologies), and 10 mM HEPES buffer (Life Technologies).

Primary AML blasts were thawed and cultured in AIM-V medium (Life Technologies) supplemented with 10% HS, 50 ng/mL stem-cell factor (SCF; PeproTech, Rocky Hill, NJ, United States) and 50 ng/mL granulocyte-colony stimulating factor (G-CSF; Hospira, Lake Forest, IL, United States) at a density of 5 × 10^5^ cells/mL in 24 well-plates. Half of medium supplemented with cytokines was replaced on days 4, 7, and 11. ATRA, AZA, and PGZ were dissolved in DMSO. Where indicated, single agent DMSO (0.1–0.3% Carl Roth, Karlsruhe, Germany; equivalent amount compared to treatment samples), ATRA (1 μM; Sigma-Aldrich, St. Louis, MO, United States), AZA (0.1 μM; Sigma-Aldrich), PGZ (5 μM; Sigma-Aldrich), or midostaurin (20 nM; Sigma-Aldrich) or drug combinations were added on day 0 of culture. Due to its short half-life AZA was added daily until day 7. Medium control contained 10% HS, SCF, and G-CSF. AML blasts were analyzed on day 14 of culture.

### Flow Cytometry and Antibodies

Flow cytometry was performed on FACSCalibur (BD Biosciences, Franklin Lakes, NJ, United States) and analyzed using FlowJo V10 software (Tree Star, Ashland, OR, United States). Relative fluorescence intensities were calculated from the MFI value of the relevant staining divided by the MFI value of the medium control sample. Fluorochrome-labeled monoclonal antibodies were anti-human CD11b (ICRF44), CD15 (HI98), CD33 (WM53), CD34 (581), CD80 (L307.4), CD83 (HB15e), CD86 (2331), CD117 (YB5.B8), CD274 (MIH1), HLA-class I (G46-2.6), HLA-DR (G46-6), and Annexin V (all BD Biosciences).

### Apoptosis Measurement

The fraction of U937 cells presenting apoptotic nuclei, among the total cell population was calculated by counting at least 300 cells in at least three different randomly selected fluorescence microscopic fields, after staining cells with the cell-permeable DNA-specific dye Hoechst 33342 directly added to the culture medium at the final concentration of 10 μg/mL (Ghibelli et al., 1995; Dini et al., 1996). Apoptosis of treated HL-60 or primary AML blasts was determined by Annexin V staining according to manufacturer’s instructions.

### Morphological Assessment of AML Cells

For morphological analysis of myeloid cell differentiation, cytospins were prepared by centrifugation (1.200 rpm, 10 min) of 5 × 10^4^ primary AML cells on microscope slides (Paul Marienfeld GmbH, Lauda-Königshofen, Germany) using a Shandon Cytospin 3 Cytocentrifuge (Thermo Fisher Scientific, Waltham, MA, United States) and air drying for 5 min. Subsequently, cytospin slides were stained for 3 min with May-Grünwald (Merck, Darmstadt, Germany) and afterward for 13 min with 25% Giemsa solution (Merck). For each condition, 200 cells were analyzed for cellular morphology and counted by two individuals independently using a Leica DMLB light microscope type 020-519.511 (Leica, Wetzlar, Germany) and Color Video Camera model MC-3289 (Horn Imaging GmbH, Aalen, Germany).

### ROS Production of Treated AML Blasts

For NBT assay (Wald et al., 2008), AML cells were seeded in 24 well-plates on day 14 of culture at a density of 1 × 10^6^ in 0.5 mL AIM-V medium supplemented with 10% HS and incubated with 1 mg/mL NBT (Sigma-Aldrich) and 200 ng/mL PMA (Sigma-Aldrich) at 37°C for 90 min. Cells were then harvested and cytospins were prepared as described above. Numbers of formazan colored cells were counted for each condition in a blinded fashion by two individuals independently.

### Transformation of *E. coli* JM109 and Preparation of Bacterial Suspension

JM109 *E. coli* bacteria were incubated with 0.25 μg enhanced GFP plasmid DNA (Schaft et al., 2006) for 30 min on ice. Following a heat shock for 45 s at 42°C, JM109 were incubated at 280 rpm, 37°C for 40 min in standard lysogeny broth (LB) medium. Finally, bacteria were plated on ampicillin agar plates and incubated over night at 37°C. Bacterial suspension was prepared with some modifications as described before (Palazzolo et al., 2005; Gille et al., 2006). Single bacterial clones were picked and incubated for 10 h at 280 rpm, 37°C in 25 mL LB medium supplemented with ampicillin (100 μg/mL; Sigma). Afterward, 500 μl of the bacterial suspension was transferred to 49.5 ml LB medium supplemented with ampicillin and again incubated at 280 rpm, 37°C until an optical density of 0.65–0.85 (λ = 600 nm). For opsonization, bacteria were pelletized (1500 rpm, 10 min) and incubated in 77% Hank’s Balanced Salt Solution supplemented with 13% PBS, 10% HS, 1 mM CaCl_2_, 0.5 mM MgCl_2_ at 280 rpm, 37°C for 30 min.

### Phagocytosis of Treated AML Blasts

Phagocytosis of GFP^+^ *E. coli* was analyzed on day 14 of AML cell culture by incubation of 2.5 × 10^5^ AML blasts with 6.25 × 10^6^ GFP-transformed *E. coli* JM109 bacteria for 10 min at 37°C in 77% Hank’s Balanced Salt Solution supplemented with 13% PBS, 10% HS, 1 mM CaCl_2_, 0.5 mM MgCl_2_. After cell harvest, cells were washed four-times with PBS and analyzed for GFP^+^ cells by flow cytometry.

### Statistical Analysis

GraphPad Prism 7.03 (GraphPad Software, San Diego, CA, United States) was used for statistical analysis. Friedman and Dunn’s multiple comparisons test was used for significance testing (non-parametric, paired analyzes). Differences were considered statistically significant for *p*-values of < 0.05.

## Results

### Anti-proliferative Effect of ATRA Is Differently Modulated by Co-treatment With PGZ and AZA in Myeloid Cell Lines

**FIGURE 1:**
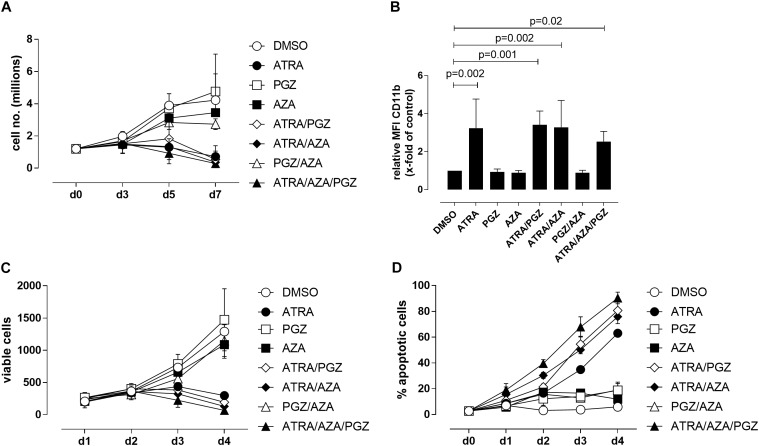
Treatment of HL-60 and U937 cells. **(A)** HL-60 cells (1.2 × 10^6^) were incubated in RPMI supplemented with 10% FCS and as indicated with DMSO (open circles), 1 μM ATRA (closed circle), 5 μM PGZ (open square), 0.1 μM AZA (closed square), ATRA/PGZ (open diamond), ATRA/AZA (closed diamond), PGZ/AZA (open triangle), or ATRA/AZA/PGZ (closed triangle). On days 3, 5, and 7 during treatment, cells were counted by trypan blue staining. Data represent means of cell numbers from three independent experiments. Error bars indicate the standard deviation. **(B)** HL-60 cells were treated as described in **(A)**. On day 5 of treatment, cells were characterized for surface-expression of the differentiation marker CD11b by flow cytometry. Shown are relative MFI values calculated by division of the MFI of antigen staining by the MFI of DMSO staining. Data represent means and standard deviations of three independent experiments. *p*-Values are shown for samples with significant differences in comparison to DMSO controls. Statistical analysis for group differences were performed using Friedman and Dunn’s multiple comparisons test. **(C,D)** U937 cells were incubated in RPMI supplemented with 10% FCS and as indicated with DMSO (open circles), 1 μM ATRA (closed circle), 5 μM PGZ (open square), 0.1 μM AZA (closed square), ATRA/PGZ (open diamond), ATRA/AZA (closed diamond), PGZ/AZA (open triangle), or ATRA/AZA/PGZ (closed triangle). From day 0 to day 4 during treatment, cells were analyzed for cell growth **(C)** and apoptosis **(D)**. Data represent means from two independent experiments. Error bars indicate the standard deviation.

We first evaluated the effects of ATRA, AZA, and PGZ as single or combined treatment on HL-60 leukemia cells (FAB M2 subtype) *in vitro*. To avoid cytotoxic effects by AZA, we used a very low dose of AZA (0.1 μM) which was at least 10-fold lower than described in other studies (Stresemann et al., 2008; Hollenbach et al., 2010). We observed that mono-treatment with ATRA, double-treatment with ATRA and pioglitazone (ATRA/PGZ) or azacitidine (ATRA/AZA) as well as the triple combination of ATRA/AZA/PGZ strongly inhibited cell growth of HL-60 leukemia cells (Figure 1A) on day 7 of culture. In contrast, cell growth of HL-60 cells treated with single agent AZA or PGZ, or their combination (AZA/PGZ) was less affected when compared to DMSO treated controls (Figure 1A). Further, we analyzed the capacity of mono- and combined ATRA, AZA, and PGZ treatment to induce cell differentiation in HL-60 cells by flow cytometric analysis of CD11b surface expression, a commonly used marker for AML differentiation (Gupta et al., 2012). Again, on day 5 of culture only treatment with ATRA alone or in combination with PGZ (ATRA/PGZ), AZA (ATRA/AZA), or AZA/PGZ (ATRA/AZA/PGZ) induced a significant upregulation of CD11b expression in HL-60 cells when compared to DMSO treated cells (Figure 1B). In clear contrast, incubation of HL-60 cells with single agent PGZ or AZA, or AZA/PGZ combination had no impact on CD11b expression, respectively (Figure 1B).

We then tested single agent and combined ATRA, AZA, and PGZ treatment on pro-monocytic U937 cells. While ATRA remains the more effective agent, AZA and PGZ display also a certain level of activity, and mostly, they both enhanced ATRA action, as shown by results from analysis of cell growth (Figure 1C) and apoptosis (Figure 1D). In conclusion, the two myeloid cell lines show similar sensitivity to ATRA, but different susceptibility to the combination treatments. Nonetheless, ATRA seems to be the key component for induction of cell death or differentiation in the analyzed cell lines.

### ATRA/AZA/PGZ Triple-Therapy Induces Apoptosis and Inhibits Cell Growth of Primary AML Blasts

**FIGURE 2:**
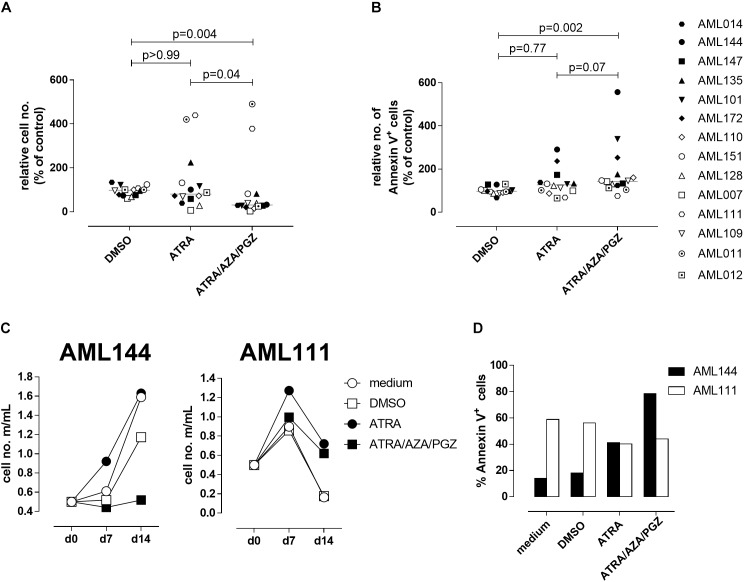
Cell growth and apoptosis of primary AML blasts. **(A,B)** Primary AML blasts (5 × 10^5^) were incubated in AIM-V medium supplemented with 10% HS, G-CSF and SCF (each 50 ng/mL) and as indicated with medium, DMSO, ATRA, or ATRA/AZA/PGZ. On 14 of treatment, cell numbers were measured by conventional trypan blue staining. **(A)** Shows cell numbers normalized to medium (*n* = 14 per group), **(B)** shows Annexin positive cells normalized to medium (medium = 100%; *n* = 14 per group). Symbols represent individual patient samples and horizontal bars mark median values. Statistical analysis for group differences were performed using Friedman and Dunn’s multiple comparisons test. **(C)** Primary AML blasts (5 × 10^5^) of patient sample AML144 (left panel) or AML111 (right panel) were incubated as described in **(A)**. On days 7 and 14 of treatment, cell numbers were measured by conventional trypan blue staining. **(D)** AML blasts as described in **(A)** were characterized by Annexin V binding 14 days after treatment. Shown are % Annexin positive cells of AML111 (white bars) and AML144 (black bars).

Since ATRA mono-treatment already induced differentiation of HL-60 cells as has also been reported by others (Gupta et al., 2012), we next analyzed the effects of ATRA/AZA/PGZ treatment on primary non-APL AML blasts isolated from 14 patients with different AML FAB and WHO subtypes (Table 1). Here, triple-treatment with ATRA/AZA/PGZ significantly inhibited growth of primary AML blasts (median of relative cell number normalized to medium 30.8%) in comparison to ATRA mono-treatment (median 80.1%; *p* = 0.04) or DMSO control (median 97.9%; *p* = 0.004) during 14 days of culture (Figure 2A). Due to limited patient material and the most striking effects of ATRA with HL-60 and U937 cells, we restricted controls to ATRA mono-treatment, DMSO, and medium control. In line with decreased proliferation, ATRA/AZA/PGZ treatment also induced an increase in apoptosis rate (median of annexin-V positive cells 143.5%), which was less distinct with ATRA (median 126.5%; *p* = 0.07) and significantly lower in DMSO controls (median 97.5%; *p* = 0.002) (Figure 2B). Most notably, effects on proliferation and apoptosis with ATRA/AZA/PGZ triple-treatment were markedly stronger than those with ATRA mono-therapy (Figures 2A,B). Of note, not all individual AML samples were sensitive to growth inhibition and apoptosis with ATRA/AZA/PGZ treatment (e.g., AML144), whereas other samples remained unaffected (e.g., AML111) (Figures 2C,D).

**Table 1:** Patients characteristics, FAB-classification, molecular and cytogenetics of primary AML samples.

Sample	FAB	WHO	Age	% Blasts in PB	Molecular genetics	Cytogenetics
AML007	M2	AM L with maturation	55–60	92	FLT3-ITD	46, XY
AML011	M5	Acute monoblastic and monocytic leukemia	85–90	95	nd	nd
AML012	M4	AML with mutated NPM1	55–60	86	FLT3-ITD NPM1 mut	46, XY
AML014	nd	AML, NOS	70–75	95	nd	nd
AML101	M4	Acute myelomonocytic leukemia	30–35	90	FLT3-ITD	46, XY
AML109	nd	AML with myelodysplasia-related changes	80–85	86	nd	46, XXder(7), t(7;11), dupl(11)
AML110	M5	AML with mutated NPM1	70–75	90	NPM1 mut	46, XY
AML111	M2	AML with biallelic mutation of CEBPA	45–50	96	Biallelic CEBPA mut	46, XY
AML128	M4	Acute myelomonocytic leukemia	75–80	93	nd	nd
AML135	M2	AML with mutated NPM1	45–50	70	FLT3-ITD NPM1 mut	nd
AML144	nd	AML with mutated NPM1	85–90	80	FLT3-ITD NPM1 mut	nd
AML147	nd	AML, NOS	70–75	86	FLT3-ITD	46Xdel(X), del(18)
AML151	MO	AML with mutated NPM1	65–70	90	FLT3-ITD NPM1 mut	46, XX
AML172	M1	AML without maturation	65–70	70	FLT3-ITD	46, XY

*CEBPA, CCAAT/enhancer-binding protein alpha; FAB, French-American-British Cooperative Group; NOS, not otherwise specified; NPM1, nucleophosmin 1; FLT3, FMS-like tyrosine kinase-3; -ITD: internal tandem duplication; nd, not determined; WHO, World Health Organization.*

### ATRA/AZA/PGZ Induces Differentiation in Primary AML Cells

**FIGURE 3:**
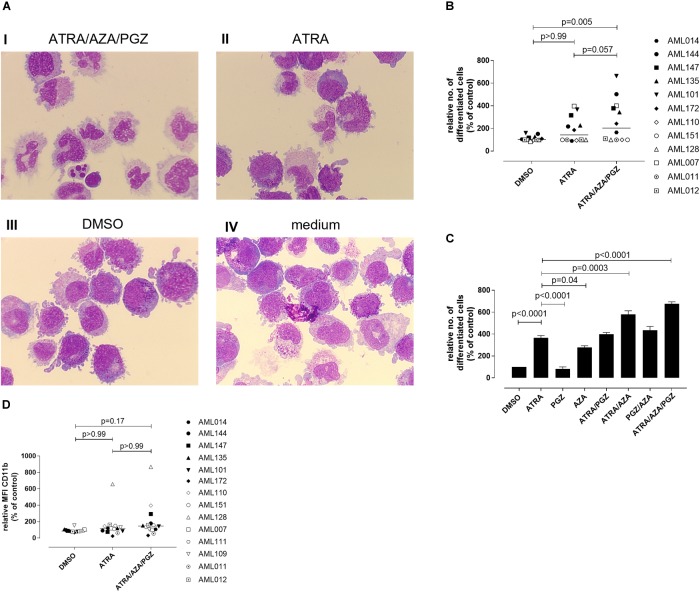
Morphological differentiation of primary AML blasts. **(A)** Primary AML blasts (5 × 10^5^) were treated as described in Figure 2. On day 14 after start of treatment, cells (5 × 10^4^) were analyzed by light microscopy after centrifugation on microscope slides and May-Grünwald and Giemsa staining. Shown are AML cells of sample AML101 after treatment with ATRA/AZA/PGZ (I), ATRA (II), DMSO (III), or medium (IV), respectively. **(B)** Summary of 12 primary AML patient’s samples on day 14 of treatment as described in **(A)**. Cells with morphological signs of differentiation were normalized to medium controls. Symbols represent individual patient samples and horizontal bars mark median values. **(C)** On day 14 of treatment with ATRA, PGZ, AZA, ATRA/AZA, ATRA/PGZ, PGZ/AZA, ATRA/AZA/PGZ, or DMSO sample AML101 was analyzed for morphological changes as described in **(A)**. Cells with morphological signs of differentiation were normalized to medium controls. Data represent means of morphological changes from two independent experiments. Error bars indicate the standard deviation. **(D)** Primary AML samples (*n* = 14) at day 14 of treatment. Shown are the relative MFIs of CD11b staining normalized to medium. Symbols represent individual patient samples and horizontal bars mark median values. Statistical analysis for group differences were performed using Friedman and Dunn’s multiple comparisons test.

We further evaluated whether ATRA/AZA/PGZ therapy promotes cell differentiation in primary AML blasts. Morphological assessment of individual AML samples on day 14 of culture demonstrated signs of differentiation as can be seen from increased and brightened cytoplasm, granular formation and altered nuclear morphology in AML cells during ATRA/AZA/PGZ therapy (e.g., AML101; Figure 3A picture I), which was less distinct with ATRA mono-treatment and absent in DMSO or medium controls (Figure 3A, pictures II–IV). In contrast, normal granulocytes or monocytes were not detected in the samples.

Whereas ATRA/AZA/PGZ triple-therapy induced morphological signs of cell differentiation in 6 of 12 analyzed AML samples, some AML samples (AML011, AML012, AML110, AML128, and AML151) remained unaffected (Figure 3B). Overall, ATRA/AZA/PGZ treatment (median normalized to medium 204.3%) induced significantly stronger differentiation in primary AML blasts than DMSO controls (median 102.6%; *p* = 0.005). Again and although not significant, effects with ATRA mono-treatment were less potent than those observed with ATRA/AZA/PGZ (median 143.5%; *p* = 0.057) (Figure 3B).

To further dissect the role of each drug, we tested single (ATRA, AZA, PGZ), double (ATRA/AZA, ATRA/PGZ, PGZ/AZA), and triple combination (ATRA/AZA/PGZ) with sample AML101 (Figure 3C). As observed in AML cell lines (Figure 1) ATRA mono-treatment had significant differentiation effects. However, also AZA mono-treatment induced significant morphological changes compared to controls (Figure 3C). Whereas ATRA/AZA combination showed an additive effect, ATRA/PGZ combination was not superior compared to ATRA mono-treatment. Again, the triple combination ATRA/AZA/PGZ induced the most pronounced differentiation effects in primary AML101 blasts.

In addition to morphological changes we investigated immunophenotypical changes of differentiation markers (CD11b, CD15, CD33, CD34, CD80, CD83, CD86, CD117, CD274, HLA-ABC, and HLA-DR). Figure 3D shows exemplified changes of CD11b expression of treated AML blasts. However, no marker showed a significant upregulation upon treatment with ATRA/AZA/PGZ compared to controls (data not shown).

### ATRA/AZA/PGZ Treatment Enhances ROS Production and Phagocytosis Activity in Primary AML Cells

**FIGURE 4:**
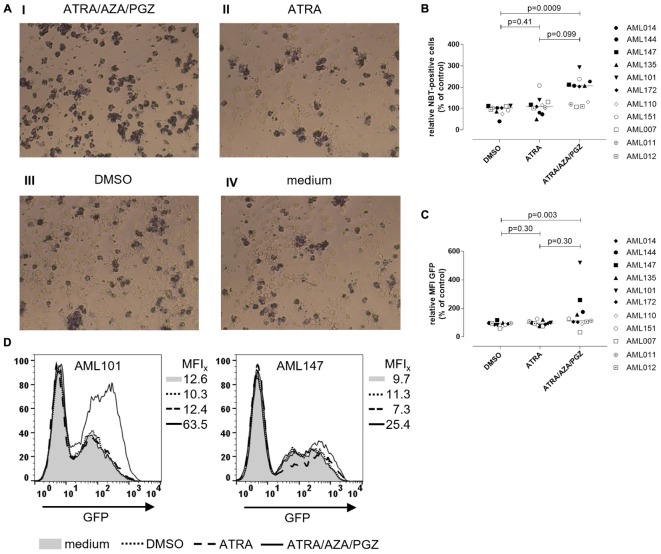
Reactive oxygen species production and phagocytosis of AML blasts. **(A)** Primary AML blasts were treated as described in Figure 2. On day 14 of treatment, 5 × 10^5^ AML blasts were incubated with NBT (1 mg/mL) and PMA (200 ng/mL) for 90 min. Subsequently, cells were centrifuged on microscope slides for light microscopy. Shown are AML blasts of sample AML101 after treatment with ATRA/AZA/PGZ (I), ATRA (II), DMSO (III), or medium (IV), respectively. **(B)** Summary of primary AML samples (*n* = 11) on day 14 days of treatment as described in **(A)**. NBT positive cells were counted in a blinded fashion and were normalized to medium. Symbols represent individual patient samples and horizontal bars mark median values. Statistical analysis for group differences were performed using Friedman and Dunn’s multiple comparisons test. **(C,D)** Primary AML blasts were treated as described in Figure 2. On day 14 of treatment, 2.5 × 10^5^ AML blasts (*n* = 11 patients) were incubated with 6.25 × 10^6^ GFP-labeled *E. coli* bacteria for 10 min. Subsequently, cells were analyzed by flow cytometry for GFP positive cells. **(C)** Summary of data (*n* = 11 AML samples) representing MFIs of GFP signal normalized to medium controls. Symbols represent individual patient samples and horizontal bars mark median values. Statistical analysis for group differences were performed using Friedman and Dunn’s multiple comparisons test. **(D)** Individual overlay plots and MFI of GFP signals measured with AML101 and AML147.

To investigate, if ATRA/AZA/PGZ treatment promotes functional differentiation in primary AML blasts, we evaluated their ability for ROS production and phagocytosis. After phagocytosis, neutrophils eliminate pathogens (e.g., bacteria) through the generation of ROS (Hong, 2017), which induces microbial DNA damage. Intracellular ROS production in stimulated cells can be measured by reduction of NBT into formazan to form an insoluble blue-black precipitate, which can be detected as intracellular color-change (Wald et al., 2008). NBT also serves as a very sensitive marker for myeloid differentiation (Gupta et al., 2012). Figure 4A shows an example of NBT staining in AML blasts after PMA stimulation. Herein, AML101 cells stained strongly positive for ROS on day 14 of ATRA/AZA/PGZ treatment (Figure 4A, picture I) which was clearly less pronounced in control treatments with ATRA, DMSO, or medium (Figure 4A, pictures II–IV). In line with morphological signs of differentiation (Figure 3), quantity of NBT staining differed between AML blasts of individual patients and was significantly higher in samples treated with ATRA/AZA/PGZ (median 207.0%) when compared to DMSO control (median 103.0%; *p* = 0.0009) (Figure 4B). Although not statistically significant, ATRA mono-treated AML samples trended to lower numbers of ROS producing cells (Figure 4B). Most notably, AML samples with higher ROS production in NBT staining (AML101, AML135, AML144, AML147) were identical with samples that showed strongest signs of morphological and immunophenotypical differentiation during ATRA/AZA/PGZ triple-therapy (Figure 3).

In addition to ROS production (Figure 4B), ATRA/AZA/PGZ combination was indeed able to induce functional differentiation in primary AML cells as shown by a significant increase of phagocytosis of GFP labeled *E. coli* in ATRA/AZA/PGZ treated samples (median 112.0%) (Figure 4C). In contrast, phagocytosis in DMSO treated samples were significantly lower (median 91.0%; *p* = 0.003) and comparable to ATRA treated cells (median 95.0%; *p* = 0.30) (Figure 4C). Again, phagocytosis was most evident in AML blasts from patient 101 (AML101) (Figure 4D, left panel).

### Effects of Midostaurin Treatment in FLT3-ITD^+^ AML Cell Lines

**FIGURE 5:**
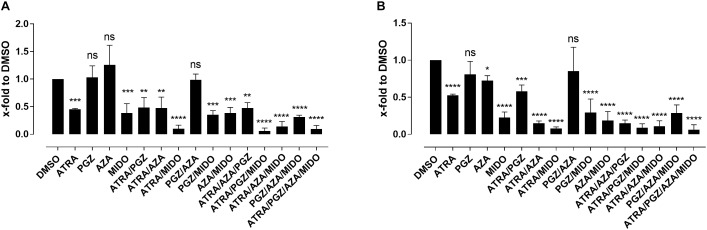
Biomodulatory treatment of AML cell lines in combination with midostaurin. **(A)** MV4-11 and **(B)** MOLM-13 cells were incubated in RPMI supplemented with 10% FCS and treated with single agent DMSO, ATRA, AZA, PGZ, MIDO or combinations thereof. On day 3 during treatment cells were counted by trypan blue staining. Data represent means of cell numbers from three independent experiments. Error bars indicate the standard deviation. Statistical analysis for group differences compared to DMSO were performed using Friedman and Dunn’s multiple comparisons test. ns *p > 0.05, ^∗^p ≤ 0.05, ^∗∗^p ≤ 0.01, ^∗∗∗^p ≤ 0.001, ^∗∗∗∗^p ≤ 0.0001*.

Since most AML samples responding to ATRA/AZA/PGZ treatment were positive for FMS-like tyrosine kinase-3 internal tandem duplication (FLT3-ITD), we analyzed the effects of the multikinase inhibitor midostaurin (MIDO) as single agent as well as in combination with ATRA, AZA, and PGZ on FLT3-ITD^+^ AML cell lines MV4-11 (Figure 5A) and MOLM-13 (Figure 5B). In line with HL-60 cells (Figure 1A), ATRA mono-treatment significantly inhibited cell growth compared to DMSO, whereas single agent AZA or PGZ had almost no effect on cell proliferation. Growth inhibitory effects of MIDO mono-treatment were comparable to those of ATRA, whereas combination therapy with ATRA/MIDO showed an additive effect on cell number leading to an almost complete cell death. Because of the strong effects of ATRA/MIDO combination leading to an almost complete cell death on day 3 of treatment, analyses of additional effects (e.g., CD11b expression, differentiation, phagocytosis) during triple or ATRA/PGZ/AZA/MIDO treatment were not valid. However, on days 1 and 2 of treatment, AZA, PGZ, or AZA/PGZ did not induce additional effects to ATRA/MIDO combination (data not shown).

## Discussion

We herein describe for the first time that a biomodulatory treatment regimen consisting of ATRA, low-dose AZA, and PGZ induces myeloid differentiation in primary AML blasts *in vitro*. Differentiated granulocyte like cells were characterized by morphological cell differentiation, expression of the myeloid differentiation markers, ROS production and most notably phagocytic activity. In addition, these observations parallel our previous data in patients with refractory AML, in which ATRA/AZA/PGZ treatment induced considerable increases of peripheral leukocyte counts while neutrophils still expressed molecular aberrations of the blast population (Thomas et al., 2015).

Most *in vitro* studies have investigated differentiation-inducing drugs such as ATRA and AZA in AML cell lines (Fujiki et al., 2012). As shown with the ATRA effect on HL-60 cells (Figure 1), however, results on cell lines may not necessarily correlate with data obtained from primary AML blasts of patient samples (Figures 2–4). Here, in comparison to ATRA mono-treatment, the combination of ATRA/AZA/PGZ was more potent to induce cell differentiation and was directly associated with increased ROS production and phagocytosis. Nevertheless, near half of 14 AML samples analyzed did not respond to ATRA/AZA/PGZ treatment and neither showed cytomorphological changes nor elevated ROS levels. However, although samples AML110 and AML128 showed a clear upregulation of CD11b (Figure 3C), we didn’t observe morphological changes or increased ROS production in those AML samples. In contrast, in other samples (e.g., AML172) we observed a downregulation of CD11b during ATRA/AZA/PGZ treatment, which might be explained by the heterogeneity of individual AML samples. This effect also indicates that CD11b upregulation did not clearly correlate with functional granulocytic differentiation in some leukemia blasts.

Differentiation of myeloid progenitors into neutrophils has been successfully achieved in patients with APL by ATRA treatment (Lo-Coco et al., 2013; Cicconi et al., 2016). In APL, treatment with ATRA and chemotherapy or more recently in combination with ATO, is highly effective and is characterized by differentiation of promyelocytes into neutrophils. Moreover, clinical data on the biomodulatory treatment regimen ATRA/ATO, meanwhile approved in low-risk APL, indicate that frequently occurring FLT3 mutations in APL are no longer of prognostic significance (Cicconi et al., 2016). Interestingly, all AML samples responding to the ATRA/AZA/PGZ combination in our study were positive for FLT3-ITD, suggesting that also AML with high-risk features may benefit from our novel ATRA/AZA/PGZ treatment approach. Since MIDO was recently approved for the treatment of FLT3 mutated AML patients, we aimed to analyze the additional effects of MIDO in combination with ATRA/PGZ/AZA treatment. As already described (Ma et al., 2016), combination of ATRA and MIDO showed additive effects (Figure 5), which could in our system not be increased by the addition of AZA and/or PGZ. However, because of the small sample number, we cannot draw any conclusions to use FLT3-ITD as a biomarker for treatment response, which should be investigated with larger sample numbers as well as in clinical trials.

Several *in vitro* studies investigated the effects of ATRA in combination with different other drugs in non-APL AML: for example, ATRA together with AZA has been shown to induce higher expression of CD11b in MLL-AF9-expressing AML cell lines (Fujiki et al., 2012). Another combination already tested in a clinical phase II trial was ATRA, AZA, and valproic acid, which led to an overall response rate of 26% in patients with refractory/relapsed as well as previously untreated AML and MDS patients (Raffoux et al., 2010). However, signs of myeloid differentiation were not described in those patients. Overall, the efficacy of ATRA in non-APL AML is poor. While addition of ATRA to intensive chemotherapy resulted in better overall survival in the NPM1 mutated subgroup (Schlenk et al., 2009, 2016), large studies by others could not confirm beneficial effects of ATRA in AML patients (Estey et al., 1999; Milligan et al., 2006; Burnett et al., 2010), which may be due to the timing of ATRA dosing and/or different patient populations. A more recent study by Verhagen et al. (2016) demonstrated that ATRA induces differentiation, decreased clonogenic capacity as well as leukemic engraftment in a poor prognostic subgroup of EVI-1 positive AML.

Since epigenetic reprogramming (e.g., by inhibition of the demethylase LSD1) has been shown to unlock the ATRA-induced therapeutic response in non-APL AML (Schenk et al., 2012), it might well be that ATRA/AZA/PGZ treatment also activates the ATRA-driven differentiation pathway by modifying the epigenetic state of leukemic cells. Indeed, the ability of the cytidine nucleoside analog AZA to induce myeloid differentiation is attributed to its activity as hypomethylating agent, even at doses lower than those used in AML (Ley et al., 1982; Taylor et al., 1984; Hollenbach et al., 2010). After incorporation of AZA into both RNA and DNA, the mechanism of action includes depletion of DNA methyltransferases, hypomethylation of DNA as well as induction of DNA damage (Kiziltepe et al., 2007; Stresemann et al., 2008; Flotho et al., 2009). While clinically achievable plasma concentrations of AZA are between 3 and 11 μM in patients with MDS treated with the clinically approved AZA dosage (Marcucci et al., 2005), effects on gene regulation in *in vitro* studies were already detected at concentration ≤ 1 μM (Stresemann et al., 2008; Hollenbach et al., 2010). However, reduced cell viability and cytotoxicity in AML cell lines have also been described at AZA concentrations of 1 μM (Hollenbach et al., 2010), which was out of action and could not be observed at concentrations of 0.1 μM in our study.

PPARγ agonists (e.g., PGZ) have also been suggested as potential modulators in myeloid leukemia by activating the transcriptional activity of target genes that control apoptosis and differentiation (Konopleva et al., 2004; Saiki et al., 2006) as well as by downregulation of the STAT5-driven quiescence of the leukemic stem cell pool (Prost et al., 2015). Furthermore, reactivation of KLF4 expression through modulation of PPARγ signaling, exerted a multifaceted tumor-suppressive effect in CDX2 positive myeloid leukemia cell lines and primary AML blasts (Faber et al., 2013), suggesting PPARγ agonists as a therapeutic modality in a large proportion of AML patients. As phosphatase and tensin homolog (PTEN) is one of the primary targets of PML/RARA in APL (Noguera et al., 2016), and loss of PTEN protein plays a critical role in determining the disease severity in myeloid malignancies (Liu et al., 2016), up-regulation of non-mutated PTEN by PGZ (Patel et al., 2001) could be an additional molecular mechanism explaining the combined activity of ATRA/AZA/PGZ. *In vivo*, administration of PPARγ agonists additionally induced BM adipogenesis, which rescued healthy hematopoietic maturation while repressing leukemia growth (Boyd et al., 2017). Together, these reports suggest a potential beneficial role of PGZ to synergize with ATRA and AZA in terms of myeloid differentiation and anti-leukemic activity in AML.

From our experiments, we cannot draw conclusions about the contribution of every single drug to the observed effects, mostly because we observed much heterogeneity in the response in various patients. Heterogeneity is confirmed by comparison of two cell lines HL-60 and U937, which show different response to AZA and PGZ. More studies will be thus required to understand the molecular determinants responsible for these differences. Detailed analyses of sample AML101 suggests that the combination ATRA/AZA might be similarly effective compared to the triple combination ATRA/AZA/PGZ (Figure 3C). However, clinical data showed only modest effects of ATRA added to hypomethylating agents (Soriano et al., 2007; Lübbert and Kuendgen, 2015), which implicates the need for additional substances like, e.g., PGZ.

## Conclusion

We show herein for the first time that biomodulatory treatment of primary AML blasts with ATRA/AZA/PGZ leads to phenotypical and functional differentiation of leukemic cells into neutrophil like cells in a significant fraction of patient samples *in vitro*. A potential clinical benefit aside from anti-leukemic activity might be a lower rate of AML- and therapy-related neutropenia and lower incidences of severe infections. These promising data with ATRA/AZA/PGZ as well as our small case series (Thomas et al., 2015) will now be verified in an ongoing phase II trial comparing ATRA, low-dose AZA, and PGZ with standard-dose AZA in elderly AML patients’ refractory to intensive induction chemotherapy (NCT02942758).

## Author Contributions

SK designed and performed the experiments, analyzed the data, and wrote the manuscript. TS contributed to the design of experiments, performed the experiments, and analyzed the data. EB and CM performed the experiments. BH supplied human samples. LG, WH, and AR discussed the data and contributed to write the manuscript. ST contributed to the design of experiments, analyzed the data, and wrote the manuscript.

## Conflict of Interest Statement

The authors declare that the research was conducted in the absence of any commercial or financial relationships that could be construed as a potential conflict of interest.

## Abbreviations

AML: acute myeloid leukemia
APL: acute promyelocytic leukemia
ATO: arsenic trioxide
ATRA: all-*trans* retinoic acid
AZA: azacytidine
CEBPA: CCAAT/enhancer-binding protein alpha
CML: chronic myeloid leukemia
DMSO: dimethyl sulfoxide
EVI-1: ecotropic viral integration site 1
FAB: French-American-British Cooperative Group
FCS: fetal calf serum
FLT3-ITD: FMS-like tyrosine kinase-3 internal tandem duplication
G-CSF: granulocyte-colony stimulating factor
GFP: green fluorescent protein
HS: human serum
MFI: median fluorescence intensity
MDS: myelodysplastic syndrome
MIDO: midostaurin
NBT: nitro blue tetrazolium
NOS: not otherwise specified
NPM1: nucleophosmin 1
PPARγ: peroxisome proliferator-activated receptor gamma
PGZ: pioglitazone
PMA: phorbol-12-myristat-13-acetat
PTEN: phosphatase and tensin homolog
ROS: reactive oxygen species
SCF: stem cell factor
WHO: World Health Organization.
